# Immune Parameters in The Prognosis and Therapy Monitoring of Cutaneous Melanoma Patients: Experience, Role, and Limitations

**DOI:** 10.1155/2013/107940

**Published:** 2013-09-19

**Authors:** Monica Neagu, Carolina Constantin, Sabina Zurac

**Affiliations:** ^1^Immunobiology Laboratory, “Victor Babes” National Institute of Pathology, 99-101 Splaiul Independentei, 050096 Bucharest, Romania; ^2^Department of Pathology, University of Medicine and Pharmacy Carol Davila, Colentina University Hospital, 21 Stefan cel Mare, 020125 Bucharest, Romania

## Abstract

Cutaneous melanoma is an immune-dependent aggressive tumour. Up to our knowledge, there are no reports regarding immune parameters monitoring in longitudinal followup of melanoma patients. We report a followup for 36 months of the immune parameters of patients diagnosed in stages I–IV. The circulatory immune parameters comprised presurgery and postsurgery immune circulating peripheral cells and circulating intercommunicating cytokines. Based on our analysis, the prototype of the intratumor inflammatory infiltrate in a melanoma with good prognosis is composed of numerous T cells CD3+, few or even absent B cells CD20+, few or absent plasma cells CD138+, and present Langerhans cells CD1a+ or langerin+. Regarding circulatory immune cells, a marker that correlates with stage is CD4+/CD8+ ratio, and its decrease clearly indicates a worse prognosis of the disease. Moreover, even in advanced stages, patients that have an increased overall survival rate prove the increase of this ratio. The decrease in the circulating B lymphocytes with stage is balanced by an increase in circulating NK cells, a phenomenon observed in stage III. Out of all the tested cytokines in the followup, IL-6 level correlated with the patient's survival, while in our study, IL-8, IL-10, and IL-12 did not correlate statistically in a significant way with overall survival, or relapse-free survival.

## 1. Introduction

Melanoma is one of the most aggressive forms of human cancer [1], although it represents only 4% of all skin cancers, it accounts for 80% of skin cancer deaths, and it is placed second after adult leukemia in terms of potential productive life-years loss [2]. The updated figures show that in 2012 only in the United States there are 76,250 new cases accompanied by 9,180 deaths due this cancer [3]. As shown in various neoplasias, tumorigenesis can be an immune-mediated disease, melanoma being sustained by a clear immune defective background. Thus, the tumour cells are not eliminated due to the activation of immune suppressive functions. Tumor initiation and progression are sustained by maintaining a chronic inflammatory state and polarized immunosuppressive regulatory cells that generate a procarcinogenesis cellular microenvironment [4].

**Figure 1:**
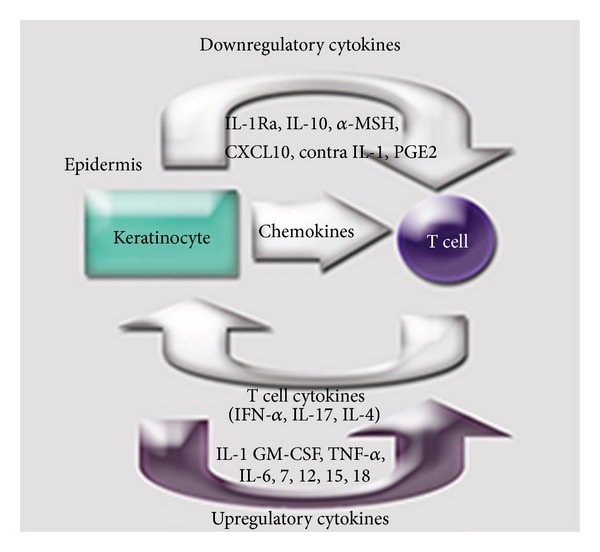
Interrelation mediated by humoral factors between keratinocytes and T cells. Keratinocytes up-regulates T cell functions by IL-1, GM-CSF, TNF-alpha, IL-6, 7, 12, 15, and 18 and down-regulates them by IL-1Ra, IL-10, *α*-MSH, CXCL10, Contra IL-1, and PGE2. T cell produces IFN-alpha, IL-17, and IL-4 that influences keratinocyte's functions. Keratinocyte's chemoattractant cytokines influences T cell trafficking: IL-1, IL-8, CCL27, CCL5, CCL17, CXCL10, MIG, IP9, and CCL20 [5].

Among the huge amount of published studies that deals with immune markers in cutaneous melanoma in the past 5 years, there are actually only tens that focus on circulatory immune markers that prove a diagnostic/prognostic value. Up to our knowledge, immune-related markers were not proficient for distinguishing benign skin disease from cutaneous melanoma. They were used for their prognostic value, whether at tumoral site or characterizing the overall immune status of the treated/untreated patient, a domain that is still insufficiently explored. Taking into account the close interrelation of the skin's resident cells with the immune cells (Figure 1) and our day-to-day experience in cutaneous melanoma, we felt that the patient's quantifiable immune parameters, namely the immune status, are important tools to be exploited in the continuous effort to improve the patient's clinical management [5]. Therefore, the present paper shows the acquired immune data in a longitudinal study in cutaneous melanoma diagnosed patients followed for 36 months during their clinical evolution. The immune parameters comprised presurgery and postsurgery immune circulating peripheral cells and the circulatory form of inter-communicating cytokines. The immune tumour infiltrating cell populations were investigated in relation to stage and further correlated with the circulatory immune cells.

## 2. Materials and Methods

### 2.1. Patients

143 patients staged according to the American Joint Committee on Cancer (AJCC) [6] were included in the study; patients entering the study in the early stages were diagnosed only with cutaneous primaries, excluding ocular melanoma or melanoma of soft parts. All melanoma patients gave written informed consent prior to their participation in this study. The standard parameters that were followed are WBC, serum LDH, S100, MIA, and routine biochemistry. Pre- and postsurgery immune parameters evaluation was performed for all patients; therefore, we registered at least two determinations of peripheral blood immune parameters.

102 patients were followed up from April 2008 to January 2012 and besides their overall clinical assessments and standard parameters, they were monitored for their immune parameters. Patients that had low WBC count displayed a mean less than 6.8 × 10^9^/liter, and lymphopenia was considered when the absolute lymphocyte count was less than 800/*μ*L.

### 2.2. Controls

270 subjects (matching gender and age) were tested for immune parameters. In the longitudinal study along with the patients, we had a group of 150 healthy subjects that agreed to follow the patient's visits; thus, we have tried to minimize the individual normal variation of the tested parameters. The selection of the constant group of donors was performed randomly on the basis of age and gender matching. 

The study had all the ethical approvals as mentioned in the acknowledgment part.

### 2.3. Peripheral Immune Cells

From peripheral blood immediately after withdraw CD3+, CD4+, CD8+, CD16, CD19+, Treg CD4+CD25+FOX3+; early activation marker CD4+/CD19+ for CD69+ were identified. Becton Dickinson kits: Multitest IMK Kit for In vitro Diagnostic Enumeration of Lymphocyte Subsets; OncoMark CD4 FITC/CD25 PE/CD3 PerCP-Cy5.5, FASTIMMUNE Assay System for early activation CD69+ marker on CD4, CD8, and CD19 were used. Biolegend kit: One Step Staining Human Treg Flow Kit (FOXP3 Alexa Fluor 488/CD25 PE/CD4 PerCP) was used. Evaluation was done with CELLQuest Software.

### 2.4. Plasma Immune-Related Cytokines

From patients' plasma immune-related cytokines/chemokines were quantified using the Luminex xMAP technology. Cytokine multiplexing for IL-1 beta; IL-6; IL-8; IL-10; IL-12 p40; and TNF-alpha kit (Human Cytokine/Chemokine Premixed 6 Plex Millipore). Acknowledging our hands-on experience, an increased number of simultaneously quantified cytokines can give false results, mainly due to the crossreactivity of labeling antibodies.

### 2.5. Tissue Samples—Immune Markers

Immunohistochemical analysis of inflammatory infiltrate within the tumor was performed on paraffin-embedded fragments of tumor harvested from surgical specimens. Several markers were investigated in order to identify T and B cells, plasma cells, and dendritic and antigen presenting cells (see Table 1). detection system used: Novolink Polymer (Leica/Novocastra) and DAB chromogen. Positive cells were analyzed in three parts of tumor: (a) main tumor mass in melanomas without regression, (b) nonregressed tumor mass in melanoma with regression, and (c) areas of regression in melanoma with regression. Results were registered as “absent” (no positive cells), “rare” cells (less than 20 positive cells/high power field HPF), and “frequent” cells (positive cells 20 or more/HPF). In case of CD1a and Langerin, only one melanoma without regression showed 23 and, respectively, 21 positive cells/HPF within the main tumor mass. For this reason, for CD1a and Langerin analysis, we used two categories “absent” and “present.” 

### 2.6. Followup and Statistics

At periodically scheduled visits, patients were clinically checked, disease progression registered by their physicians, and bled for monitoring their immune parameters. For statistical analyses, mean values ± SD was performed, a two-tailed Student's *t*-test was used, and significance was defined as *P* < 0.05 when comparing various groups or parameters.

## 3. Results and Discussion 

### 3.1. Melanoma Patients' Characterization

The investigated groups comprised subjects diagnosed in all AJCC stages (Table 2). The majority of the investigated patients were diagnosed in stages I and II (74% out of the total number of patients), while the rest of patients (26%) were diagnosed at presentation in stages III and IV. The overall age range of investigated patients was 18–89 years with an overall predominance in female patients. Out of the total group of patients, 13 were registered at presentation with lymphopenia (absolute lymphocyte count < 800/*μ*L). In all the patients, the circulating percentage of lymphocytes population/subpopulations was calculated upon the absolute lymphocyte counts.

### 3.2. Immune Parameters before Melanoma Treatment

#### 3.2.1. Peripheral Immune Cells

Besides standard evaluation (laboratory and clinical parameters), patients were evaluated for peripheral immune parameters before surgery/treatment and 1 month after. Taking into account our experience regarding postsurgery immediately tested parameters (existence of normal regenerative and inflammation processes), we did not consider these data meaningful in the context of the presented results. Thus, patients that agreed to be followed were tested one month after surgery and thereafter followed up for two-three years regularly. 

**Figure 2:**
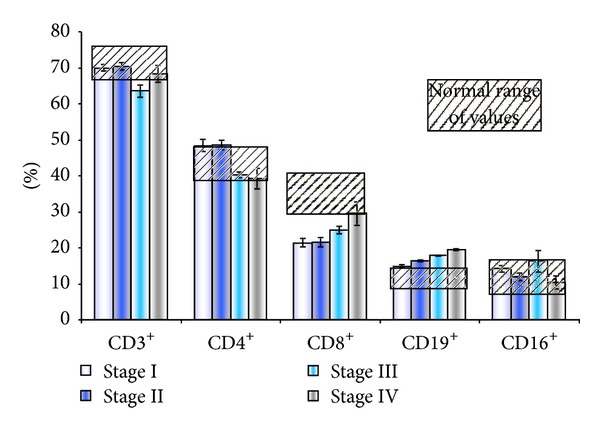
Peripheral blood lymphocytes in cutaneous melanoma patients in comparison to normal ranges (% from CD45+ pan leukocytic antigen)—CD8+ in stages I and II compared to normal values *P* < 0.005, CD19+ in stages III and IV compared to normal values *P* < 0.005.

Before tumour surgery or any other treatment for advanced melanoma stages, patients had an interesting immune cellular pattern (Figure 2). The total circulating T lymphocytes do not display abnormal values no matter the stage. The circulating CD4+ T cells have a tendency to decrease in more advanced stages but are not statistically different from normal ranges. The most interesting pattern was found in stages I and II where there is statistically significant lower percentage of CD8+CD3+ T lymphocytes and normal values for B CD19+ and NK cells. In advanced stages (III and IV), normal CD8+ T and NK cells were registered, while statistically higher B lymphocytes were registered. In advanced stages there is a decrease of the classic T-CD4+/T-CD8+ ratio [5], based mainly on the increase of the circulating T-CD8+ subpopulation. We have additionally investigated the total serum immunoglobulins (Igs), and we found no correlation between the levels of the circulating Igs, classes, or total Igs with the high circulating percentage of B lymphocytes. The levels of circulating B lymphocytes showed a reverse correlation with the level of circulating NK cells, as previously reported by us [7]. 

**Figure 3:**
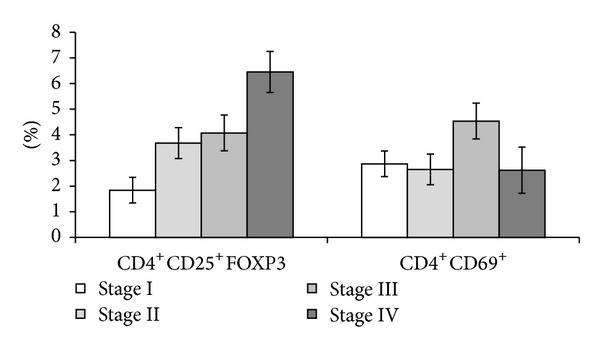
Circulating CD4+ T cells in cutaneous melanoma patients.

We have noticed that in more advanced stages there is a doubling of cells with CD4+CD25+FOXP3 phenotype (Figure 3). Interestingly, we found the percentage of circulating CD4+ T lymphocytes expressing early activation marker CD69+ slightly higher in stage III and no statistical differences in early or late stage patients. Tregs (CD4+CD25+FOXP3+) were registered in normal donors in the range of 2–4% out of the total circulating CD4+ T cells. Up to our knowledge, there is no reporting of the circulating Tregs in normal Romanian population. In international references, the reported values of circulating Tregs can range from 4.4 to 13.0% out of the total circulating CD4+ T cells in one study [8], while 1 to 6% in another [9]. The differences with the already published results regarding normal circulatory Tregs can be sustained by different types of detection methods or even by differences among cellular tested population of healthy individuals.

Circulating Tregs in patients diagnosed in stages I–III when compared to normal values do not display statistically significant differences, while we have obtained an increase in the circulatory Tregs in stage IV. 

An interesting finding was in stage III where we have registered an activation pattern for circulating CD4+ T cells, while the other stages did not register any statistical differences. 

Early activation antigen on T cells, CD69, is expressed transiently, and it was reported that in the periphery, it down regulates sphingosine-1-phosphate. This leads to the inhibition of lymphocyte circulating from the lymph node and the retention of T lymphocytes in the lymph node [10, 11]. These arguments come in favor of the detection in stage III (before any treatment) of a statistically relevant CD4+CD69+ circulatory population, that is prone to elicit an antitumoral response in an already invaded lymph node. However, in a study published several years ago, it was shown that before therapy, patients that had low proportions of circulating CD3+CD4+CD69+ and CD3+CD56+ proved an increased interval of disease-free survival compared to those with high proportions. This study strengthened the prognostic potential of circulating T cells displaying the early activation marker in melanoma [12].

#### 3.2.2. Circulating Levels of Cytokines

**Figure 4:**
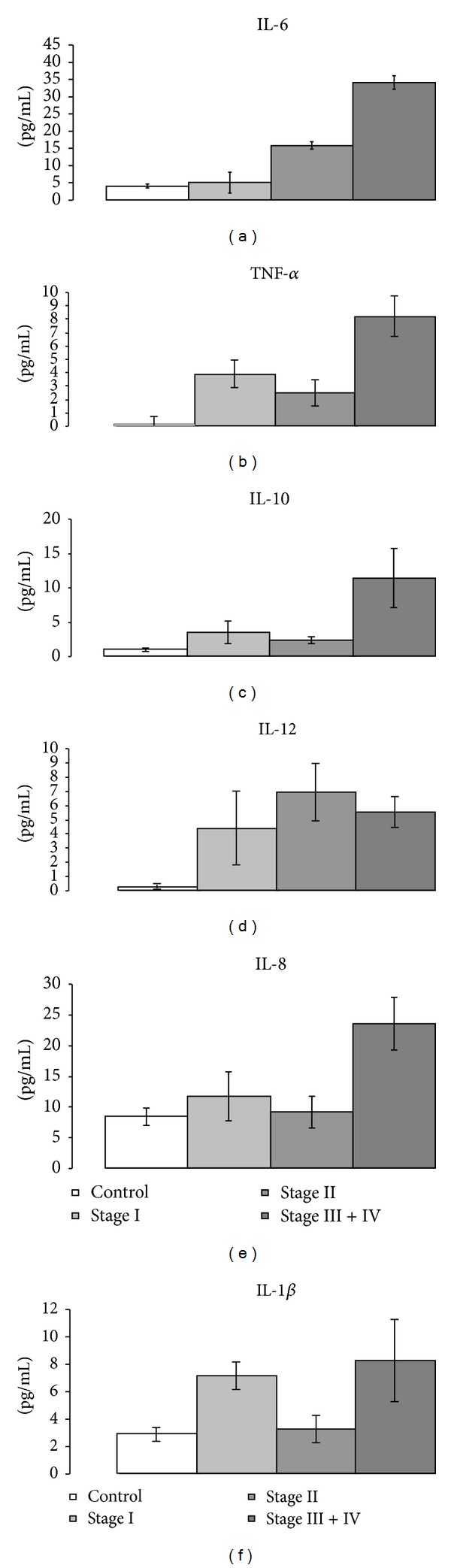
Plasma level of IL-6 (a), TNF-*α* (b), IL-10 (c), IL-12 (d), IL-8 (e), and IL-1*β* (f) in melanoma patients compared to control.

Testing concomitantly the panel of circulating cytokines/chemokines from the diagnosed patients, we have seen several statistically altered levels compared to controls. Circulating IL-6 (Figure 4(a)) is statistically different from controls only in advanced stages (*P* < 0.005), while for stage II, we have evaluated a slight statistically significant increase in the circulatory IL-6 (*P* = 0.5). It is known that skin cells, like keratinocytes, produce cytokines that upregulate T-cell functions [5]. Finding an increased circulatory IL-6 can account for the immune system upregulation done by keratinocytes to enhance the T cells antitumoral activity. The same explanation can account for the circulatory high values of TNF-alpha. All the stages displayed statistically higher values compared to controls (Figure 4(b)). As expected, the highest circulatory TNF level was found in advanced stages, a mean of two-fold increase compared to early stages (I and II). Although not statistically different, we can note an interesting elevation of circulatory TNF in stage I compared to stage II. 

Circulatory IL-10 was found strongly elevated in late stages of melanoma (Figure 4(c)) and had a good positive correlation (PC = 0.94) with the circulatory elevated T lymphocytes CD4+CD25+ with FOXP3 expression (Figure 3). In a previously published study focusing on metastatic melanoma patients, circulating Tregs were reported as specific for tumour antigens like gp100, TRP1 NY-ESO-1, survivin, and so on. In that study, Tregs from peripheral blood mononuclear cells (PBMC) cultures were found proliferating and producing preferentially IL-10 [13]. Thus, the correlation between the high plasma values of IL-10 found by us in melanoma advanced stages can account for the increased circulating Tregs. Although a previous study has stated that IL-10 was not detectable in melanoma serum patients [14], we do not exclude the existence of differences regarding the multiplexing method *versus* classical ELISA; moreover, we have used plasma that offers an increased availability for detecting lower concentration of cytokines.

Circulatory IL-12 was found undoubtedly high in all patients no matter the diagnosis stage (Figure 4(d)). Note that we have detected IL-12 subunit p40 in the patients' plasma and that IL-12p40 is known as a component of the bioactive interleukins IL-12 and IL-23 [15]. IL-12 is secreted by a plethora of cells like antigen presenting cells (monocyte/macrophages, dendritic cells, and B lymphocytes), mast cells, and nonimmune cells such as keratinocytes. In addition, IL-12 has receptors on NK and T lymphocytes that increase their proliferation and cytotoxic capacity [16]. Thus, the finding that IL-12 is enhanced in the circulation of melanoma patients can be accounted for by an active immune response. Although not statistically different, we can recognize an increase of IL-12 level in early melanoma stages, followed by a decrease in more advanced ones. 

IL-8 present in patients' plasma is statistically elevated only in advanced melanoma stages, a 2.5-fold increase compared to controls (Figure 4(d)). Thoroughly revised by Dewing et al. [17], IL-8 has a chemokine function, actively secreted by macrophages, endothelial cells, and tumour cells, favoring metastatic processes. An increase of this chemokine in the later stages of disease was somewhat to be expected. Only patients in stage I showed a slight correlation of IL-8 level with the circulatory percentage of CD16+ and CD8+ (PC = 0.75) and no other significant correlation. Overall, high IL-8 levels have been registered in patients with metastatic melanoma, and a decrease of serum IL-8 level has been described as a result of chemotherapy or immune therapy [18, 19].

One of the most interesting circulatory cytokines in terms of the plasma level in association to staging is IL-1beta. We have chosen to focus on the beta form of IL-1 due to various reasons: some human melanoma cells can spontaneously produce functional IL-1beta [20], the paracrine function of IL-1beta is known [21], and its involvement in tumour growth and inflammation is well proven [22]. Thus, stage I has a two fold increase in the circulating level compared to control, while stage II has no difference from the normal values. Advanced stages (III and IV) display a circulating IL-1beta at the same level as stage I. Previous data have shown that at the tumoral level, the expression of IL-1beta increases in primary tumour versus normal/benign tissue and increases once more in the metastatic tumour [20]. Therefore, we have identified the circulating levels of IL-1beta that can indicate the evolution of the metastatic processes. The patients that we have enrolled were not tested for the existence of BRAF (V600E) mutation in the excised primary or metastatic tumours. Knowing that the expression of BRAF (V600E) can induce both the transcription of IL-1alpha and beta in melanocytes and melanoma cell lines [23], we can account that, at least for a part of the patients, the enhanced level of circulatory cytokine can be due to BRAF (V600E) mutation. 

We found very low levels of IFN-gamma with no statistical relevance between either of the investigated groups, matching an older study showing that the levels of IFN-gamma were less elevated in patients while detectable IFN-gamma patients had higher risk for recurrence [24]. 

#### 3.2.3. Tumour Inflammatory Infiltrate

**Figure 5:**
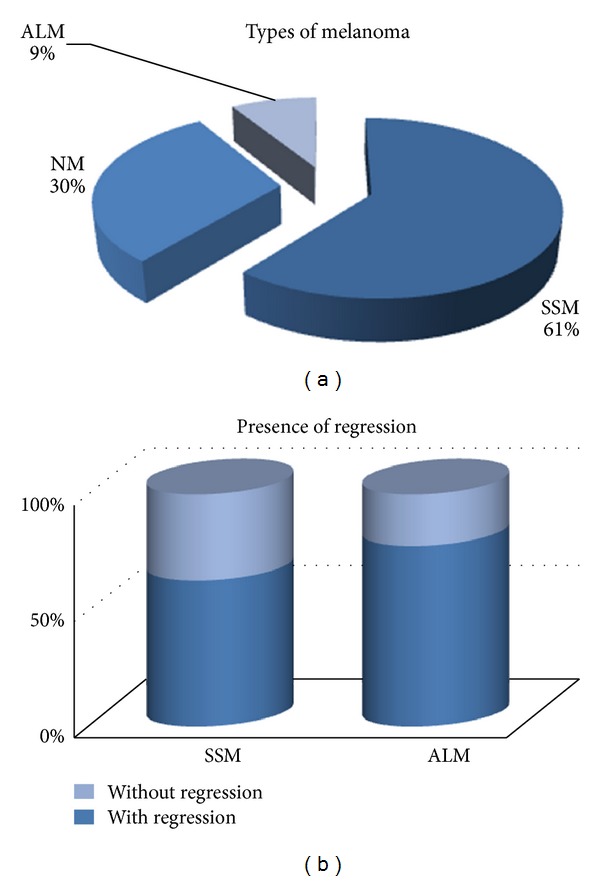
(a) Types of melanoma. (b) Presence of regression according to melanoma type.

Our lot includes 62 superficial spreading melanomas (SSMs), 31 nodular melanomas (NMs) and 9 acral-lentiginous melanomas (ALMs) (Figure 5(a)). Some SSM and ALM cases presented regression (64.79%), in different proportion for each type of tumor (SSM: 62.90%; ALM: 77.78%) (Figure 5(b)). We analyzed the inflammatory infiltrate separately, in both regressed and nonregressed component, due to the obvious differences in appearance and distribution of inflammatory cells in these areas. 

 Inflammatory infiltrate mainly consisted in T lymphocytes (CD3+). When analyzing the presence of CD3+ cells within tumor mass (quantified as absent, rare, and frequent) versus tumor-infiltrating lymphocytes (TIL) (quantified as absent, nonbrisk, and brisk), we found a perfect match between these categories. These findings were similar with other authors' data [25, 26].

**Figure 6:**
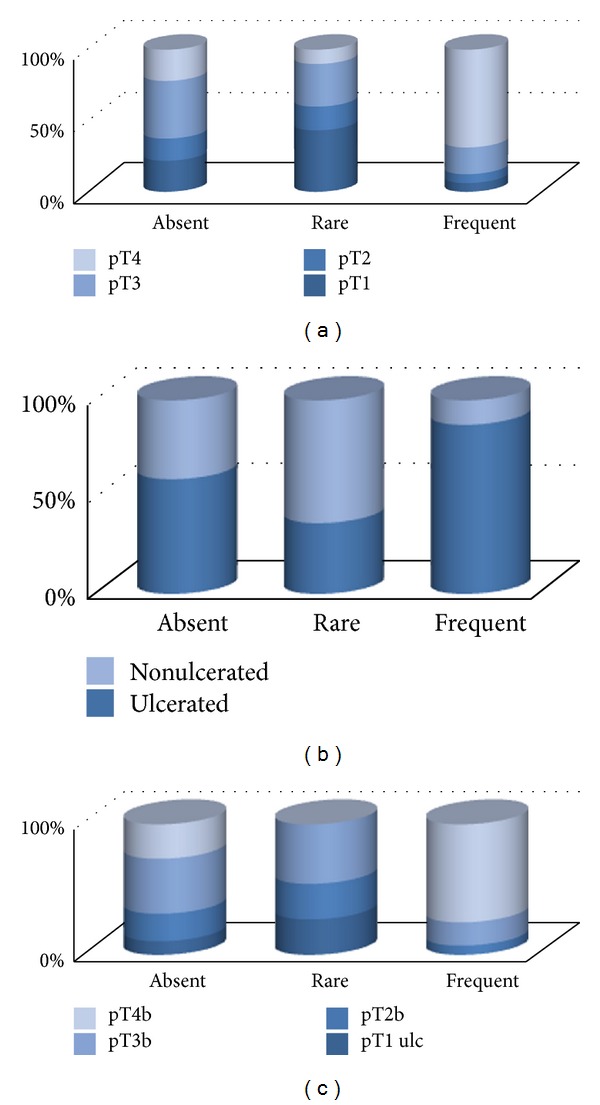
(a) Correlation between CD3+ cells density and pT level. (b) Correlation between CD3+ cells density and ulceration. (c) Correlation between CD3+ cells density and pT level in ulcerated tumors.

We identified a significant association between high pT level and the presence of frequent CD3+ T cells (*P*_frequent  versus  absent  CD3+_ = 0.002; *P*_frequent  versus  absent  CD3+_ < 0.001) (Figure 6(a)) and ulceration and presence of frequent CD3+ cells (*P*_frequent  versus  absent  CD3+_ = 0.01; *P*_frequent  versus  absent  CD3+_ < 0.001) (Figure 6(b)). Nonulcerated tumors have similar distributions of CD3+ cells irrespective of pT level; significantly more numerous cases with ulceration presented frequent CD3+ cells in association with high pT levels (*P*_frequent  versus  absent  CD3+_ = 0.007; *P*_frequent  versus  absent  CD3+_ = 0.0001) (Figure 6(c)). These findings are surprising, considering the overall favorable prognostic significance associated with brisk TIL [27–33], our data indicates the presence of abundant TILs within thick ulcerated tumors (unfavorable prognosis). More likely, our results represent the reflection of a normal increasing of the inflammatory infiltrate within an ulcerated tumor as a physiologic reaction to ulceration.

All regressed areas presented very numerous CD3+ cells (over 200 cells/HPF). No significant differences occurred in the density of CD3+ cells within the tumor mass between nonregressed areas in cases with regression and cases without regression, irrespective of tumor type (SSM and/or ALM).

CD5 presented a similar distribution as CD3. There were some differences when analyzing CD7 (overall tendency of loosing CD7 expression in inflammatory infiltrate both intratumor and in regression areas), but the differences were minor.

Analyzing CD4+ and CD8+ T cells, we identified a slight predominance of CD4+ cells in most cases (CD4 : CD8 > 1 : 1) without differences when correlating with pT level, ulceration, or regression. Also, there was a slight tendency of increasing CD4+ cells number in cases with higher pT levels. There was no correlation between the data obtained by evaluating circulating immune cells and tumor associated ones. This apparent inconsistency between specific T cells found both in the same patients' tumor tissue and blood is well documented [34].

**Figure 7:**
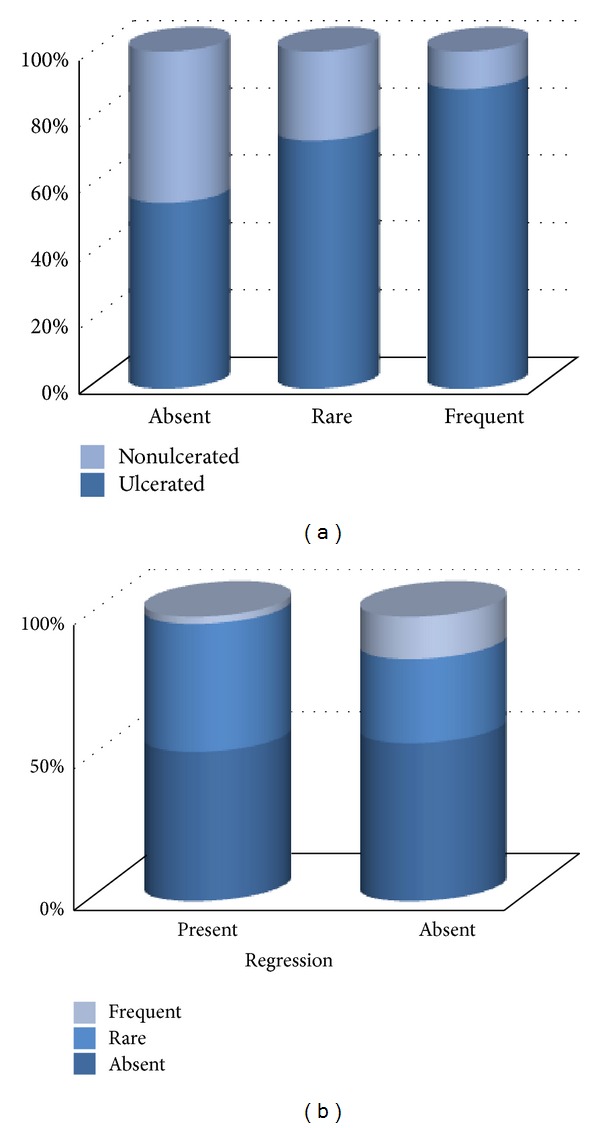
(a) Correlation between density of CD20+ cells and presence of ulceration. (b) Correlation between density of CD20+ cells within the tumor mass and presence of regression.

We analyzed the presence of B lymphocytes (CD20+). We recorded an increased number of B cells in pT4 tumors and almost statistically significant predominance of B cells in ulcerated tumors (*P* = 0.06) (Figure 7(a)). Less numerous CD20+ cells were present in the nonregressed component of tumor with regression than in tumors without regression, but the results had no statistical significance (*P* = 0.07) (Figure 7(b)). The presence of B cells within TIL was previously identified [35] in other tumors being correlated with better prognosis [36]. In our cases, the presence of more numerous B cells in ulcerated tumors may be secondary to ulceration (as part of subsequent inflammatory reaction) and therefore should not be regarded as an indicator of bad prognosis. 

**Figure 8:**
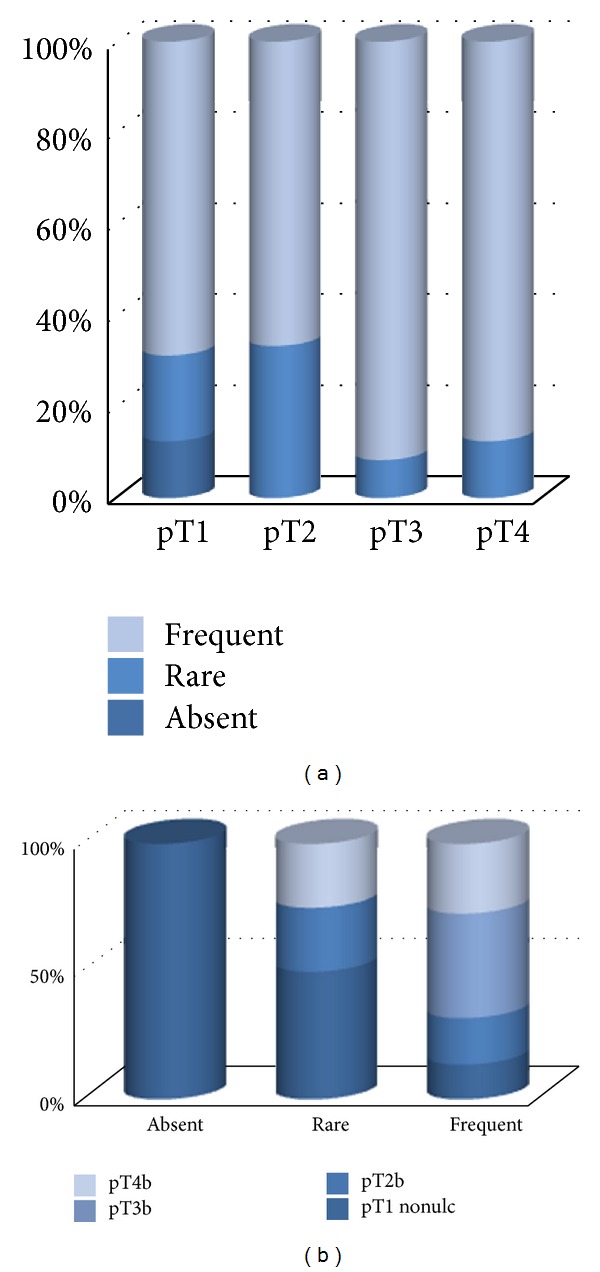
(a) Correlations between the presence of CD138+ cells and pT level. (b) Correlations between the presence of CD138+ cells and pT level in ulcerated tumors.

CD138+ cells (plasma cells) were present in almost all areas of regression (95.23%) and, when present, they were frequent, irrespective of the type of regression or ulceration. More numerous CD138+ cells were present in areas of regression in tumors with high pT, especially in ulcerated ones, but the data lacks statistical significance (Figures 8(a) and 8(b)).

CD23+ cells were absent in tumors without regression; they were present in 34.88% of cases in areas of regression and in only two cases in nonregressed areas. 

**Figure 9:**
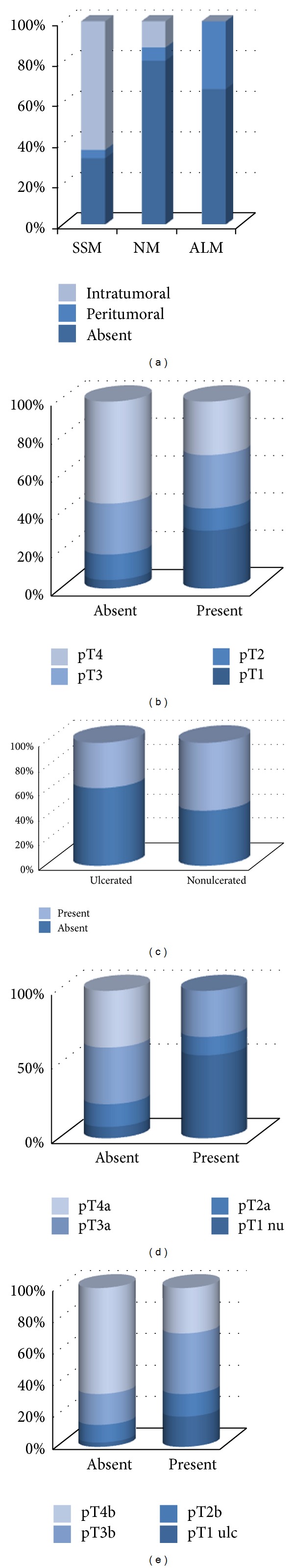
(a) The presence of Langerhans cells within tumor mass correlated with tumor type. (b) The presence of CD1a+ cells within tumor mass correlated with pT level. (c) The presence of CD1a+ cells correlated with the presence of ulceration. (d) The presence of CD1a+ cells correlated with pT level in nonulcerated tumors. (e) The presence of CD1a+ cells correlated with pT level in ulcerated tumors.

Interesting results occurred when evaluating the presence of Langerhans cells (LC) (CD1a+ Langerin+). Within the tumor mass, LC was either absent or scarce (isolated cells, less than 20 cells/HPF in all cases but one). SSM presented LC within tumor mass more frequent than NM (*P* < 0.001) or ALM (*P* = 0.003) (Figure 9(a)). The presence of LC within tumor mass correlates with Breslow index (higher pT level, more probable absence of LC—*P* = 0.007) (Figure 9(b)) but not with ulceration (more numerous non-ulcerated tumors have LC but not statistically significant) (Figure 9(c)). However, when correlating LC and pT level separately in ulcerated and nonulcerated tumors, we identified their presence in thinner tumors (Figure 9(d) nonulcerated tumors, *P* = 0.01, and Figure 9(e) ulcerated tumors, *P* = 0.02). The presence of LC within the tumor is associated with the factors of better prognosis (thinner tumors), most likely these cells being involved in antitumor host defense by presenting antigens to CD8+ cells [37, 38].

### 3.3. Immune Parameters Evaluated in Longitudinal Study

Reports of long-term monitoring of immune parameters do not abound in this type of cancer. We have studied in the longitudinal study 102 patients, 74 in stages I and II and 28 in stages III and IV. The overall survival rate of patients at the time of publication is as follows: 100% for stage I, 92% for stage II, 70% for stage III, and 30% for stage IV. Apart from primary tumour surgery with wide margin excision, stages I and II did not receive any other therapy intervention. Stage III was surgically treated for tumour and lymph nodes resection, and postsurgery therapy consisted of high doses of IFN-alpha-2b and/or dacarbazine-based combination chemotherapy regimens. Stage IV patients were treated with surgery reintervention if metastasis was approachable, and postsurgery treatment included dacarbazine or temozolomide-based combination chemotherapy regimens. None of the presented patients had any kind of vaccine-based immunotherapy. 

**Figure 10:**
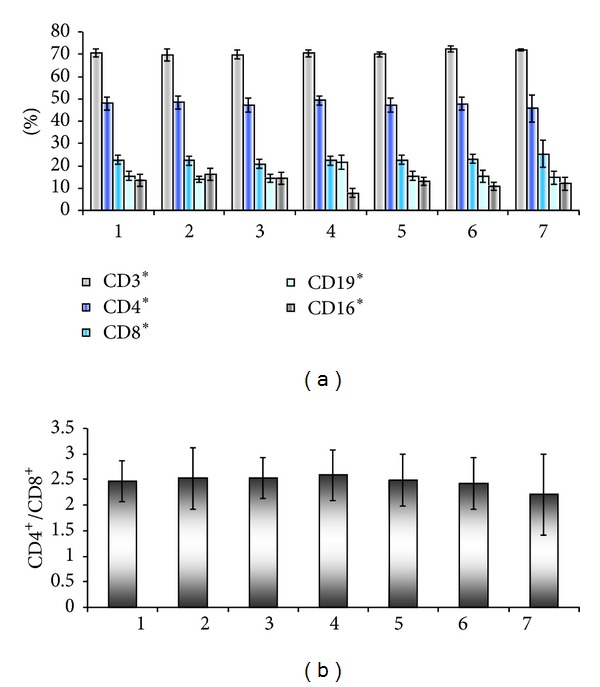
Peripheral blood immune populations evaluated in stage I patients before surgery (1) and 36 months followup in 6 visits (2–7). Percentage of circulating immune subpopulations (a) and CD4/CD8 ratio (b).

Patients that were studied in dynamics in the longitudinal evaluation showed various patterns. In stage I (Figure 10), there is an unchanged level of CD3+ in peripheral circulation (a) and a tendency for the CD4/CD8 ratio to drop after 3 years of followup (b).

**Figure 11:**
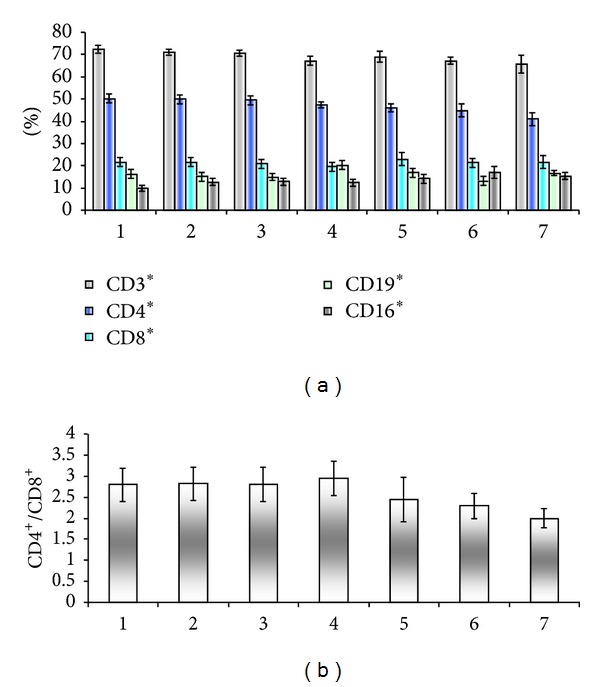
Peripheral blood immune populations evaluated in patients stage II before surgery (1) and 36 months followup in 6 visits (2–7). Percentage of circulating immune subpopulations (a) and CD4/CD8 ratio (b).

The registered tendency to drop the CD4/CD8 ratio in stage I (Figure 10(b)) is more evident in stage II (Figures 11(a) and 11(b)).

**Figure 12:**
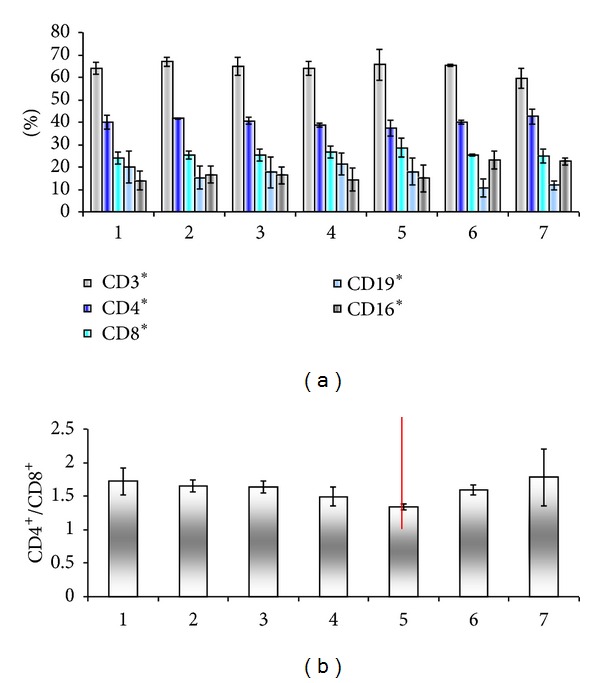
Peripheral blood immune populations evaluated in patients stage III before surgery/therapy (1) and 36 months followup in 6 visits (2–7). Percentage of circulating immune subpopulations (a) and CD4/CD8 ratio (b).

In stage III, the same decrease of CD4/CD8 ratio was identified (Figures 12(a) and 12(b)), and then an increase of this ratio (marked in red line). This marking identifies the time when patients diagnosed at presentation with the disease started to exit the study due to natural causes: death, impossibility to follow up due to the low quality of life, and so on.

Moreover, it is noticeable that the SD of the results increased as the actual number of enrolled patients drops. The decrease in circulating B lymphocytes is balanced by an increase in circulating NK cells (Figure 12(a)). We can note this regulatory mechanism between immune circulating populations in stage III mechanism that probably compensates between the two arms of the immune response.

**Figure 13:**
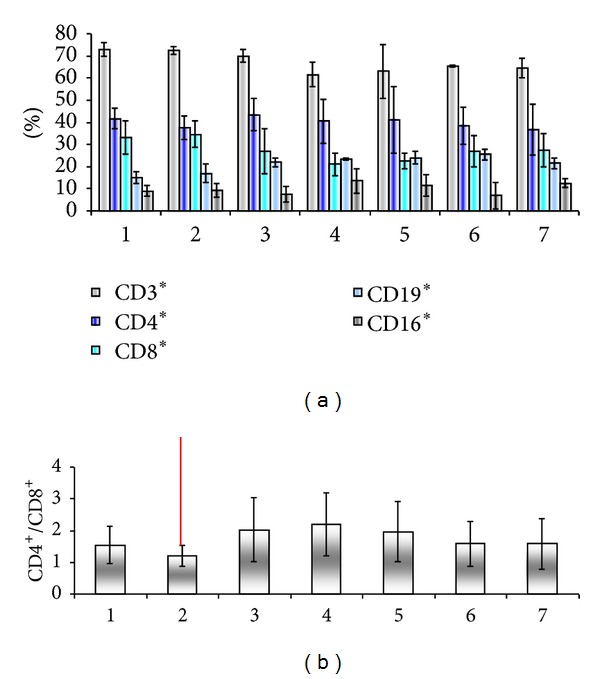
Peripheral blood immune populations evaluated in patients stage IV before surgery/therapy (1) and 36 months followup in 6 visits (2–7). Percentage of circulating immune subpopulations (a) and CD4/CD8 ratio (b).

In stage IV patients at presentation (Figure 13(b)), the patients' cell number drops earlier in the followup, and it is more obvious the increase of CD4/CD8 ratio for patients that survive more than a few months. We can postulate that the shown increased SD resides in the actual number of enrolled patients that is decreased. The compensatory mechanism between B and NK is not as obvious as in stage III probably due to a generally impaired immune response.

**Figure 14:**
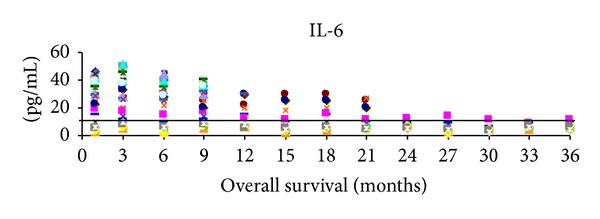
Plasma level of IL-6 for all the investigated patients during follow-up, cross-line depicts the 10 pg/mL cut-off.

During followup, the plasma IL-6 proved to be a good prognosticator of the overall survival. When we applied for IL-6 the 10 pg/mL cutoff value that was obtained from the first quartile of the values registered at first presentation in all patients, a clear correspondence of IL-6 level with survival appeared (Figure 14). A study published by the Italian Melanoma Intergroup evaluating chemotherapy versus biochemotherapy showed that higher values of IL-6 correlated with a worse survival [39]. An experimental mouse model has demonstrated that IL-6 is involved in both the development and progression of skin melanoma [40]. Furthermore, a review [41] summarized recent clinical studies focusing on IL-6 and melanoma. In the light of our findings, it could be possible to stratify patients with high IL-6 levels to be treated with an anti-IL-6 receptor monoclonal antibody like tocilizumab. 

For IL-12 found statistically elevated in all the patients no matter the stage, we did not find these clear-cut results as we did in IL-6, although the previously cited study by the Italian Melanoma Intergroup indicated IL-12 as a good prognosticator. The same case was for IL-8 and IL-10, meaning no correlation with overall survival or relapse-free survival.

## 4. Conclusion—“The Good, the Bad, and the Insensitive”

Immunohistochemical phenotype of TIL in melanoma is comparable to the circulating immune cells without a perfect overlapping, with some differences occurring mainly due to the systemic immune response depicted in the blood circulating cells, the lymph node trafficking to and from the tumor, and so on.

Intratumoral CD3+ cells were frequent in advanced stages and ulcerated tumors, more likely as an inflammatory response towards ulceration and not a host defense against tumor. In regressed areas, we found both CD4+ and CD8+ cells with a CD4/CD8 supraunitary ratio but no correlation with pT level in presence of ulceration or regression. 

Similarly to peripheral blood, more numerous B cells were present in advanced stages and/or ulcerated cases. Langerhans cells were not frequent within the tumor mass but, when present, they were associated with the histopathologic parameters of proven favorable prognosis such as thin tumor, irrespective of absence or presence of ulceration. Based on our analysis, the prototype of the intratumor inflammatory infiltrate in a melanoma with good prognosis is composed of numerous T cells CD3+, few/absent B cells CD20+, few/absent plasma cells, and present Langerhans cells.

When drawing the circulatory immune parameters of a melanoma patient first of all, testing the absolute count of lymphocytes will provide the correct data for detecting the actual circulating subpopulations. CD3+ total T lymphocytes is a parameter that will change during the followup just in advanced stages and will not give an early prognosticator, while the CD4/CD8 ratio will indicate the evolution of disease and will prognosticate the overall survival of the patient, no matter the stage and the applied therapy. We found an increase only in stage III of the circulating percentage of T cells with CD4+CD69+ phenotype indicating a lymph node-related antitumoral activity. We had no correlation to the other stages or to the clinical evolution of patient, although there are statements that pretreatment percentages of circulating CD3+CD4+CD69+ cells can be an independent prognostic factor for overall survival [42]. Peripheral Tregs increase with stage, but we could not establish a correlation between the degree of metastasis and the percentages of circulatory Tregs as previously published [43]. Advanced stages show statistically higher circulating CD19+ B lymphocytes with no increase in plasma level of total and/or subclasses Igs. There is a negative correlation between the level of circulating B lymphocytes and NK cells in melanoma patients.

Circulatory cytokines have different patterns matching the cutaneous melanoma stages. Thus, IL-6 increases with stage, as do TNF-alpha and IL-8. We found plasma IL-6 strongly positively correlated with other serum markers tested in our patients, like S100 and MIA.

IL-6 can pinpoint the overall survival of the patient, and the circulating levels of IL-1beta can indicate the evolving of metastatic processes. Some other cytokines like TNF-alpha, IL-8, and IL-10 increased only in advanced stage not proving, at least in our group, any discrimination power for early stages. Out of all the tested cytokines in the followup, IL-6 level correlated with the patient's survival, while IL-8, IL-10, and IL-12 did not correlate with overall survival or relapse-free survival. 

A panel of circulatory immune markers can complete the immune status of the patient and can bring added value to the overall prognosis of the patient and thus direct/redirect the therapy choice. The future lies within establishing low-cost, affordable/available, and easily reproducible assays that will complete the preclinical parameters of the patient.

## Figures and Tables

**Table 1:** Immunohistochemical markers.

Antibody	Source	Clone	Host	Working dilution	Pretreatment*
CD3	Leica/Novocastra	PS1	Mouse	0.3/200	HIER, buffer citrate, and pH 6
CD5	idem	4C7	idem	1/200	idem
CD4	idem	4B12	idem	5/200	idem
CD23	idem	1B12	idem	RTU	idem
Langerin	idem	12D6	idem	0.5/200	idem
CD1a	idem	JPM30	idem	2/160	idem
CD7	Thermo Scientific/Neomarkers	272	idem	RTU	idem
CD8	idem	C8/144B	idem	2/200	idem
CD20	Cell Marque	L26		RTU	idem
CD138	Invitrogen	—	Rabbit	1/200	HIER, EDTA citrate, and pH 8

**Table 2:** Cutaneous melanoma patients' characteristics at presentation, percentage of patients diagnosed in different stages, age, and gender.

Stage at presentation (% out of the total group)	I (34%)	II (40%)	III (15%)	IV (11%)
Age (mean ± SD years)	57 ± 3	51 ± 4	59 ± 1	67 ± 4
Age range (years)	18–72	20–68	22–89	35–70
Gender (% women)	89%	72%	33%	66%

## Abbreviations

AJCC: American Joint Committee on Cancer
ALMs: Acral-lentiginous melanoma
Alpha-MSH: Alpha-Melanocyte-stimulating hormone
CCL: Chemokine ligands
CXCL: Chemokine (C-X-C motif) ligand
GM-CSF: Granulocyte macrophage colony-stimulating factor
gp100: Glycoprotein100
HIER: Heat induced epitope retrieval
HPF: High power field
IFN: Interferon
Igs: Immunoglobulins
IL: Interleukin
IP9: CXCL11
LDH: Lactate dehydrogenase
MIA: Melanoma inhibitory activity
MIG: Monokine induced by gamma interferon
Nk: Natural killer cells
NM: Nodular melanomas
NY-ESO-1: New York esophageal squamous cell Carcinoma 1
PBMC: Peripheral blood mononuclear cells
PGE2: Prostaglandin E2
S100: Calcium-binding protein soluble in 100% saturated ammonium sulfate
SSM: Superficial spreading melanoma
TIL: Tumor infiltrating lymphocytes
TNF-alpha: Tumor necrosis factor alpha
Treg: T regulatory cells
TRP1: Tyrosinase-related protein 1
WBC: White blood cells.
